# Predictors of blood pressure response to ultrasound renal denervation in the RADIANCE-HTN SOLO study

**DOI:** 10.1038/s41371-021-00547-y

**Published:** 2021-05-24

**Authors:** Manish Saxena, Roland E. Schmieder, Ajay J. Kirtane, Felix Mahfoud, Joost Daemen, Jan Basile, Philipp Lurz, Philippe Gosse, Kintur Sanghvi, Naomi D. L. Fisher, Lars C. Rump, Atul Pathak, Peter J. Blankestijn, Anthony Mathur, Yale Wang, Michael A. Weber, Andrew S. P. Sharp, Michael J. Bloch, Neil C. Barman, Lisa Claude, Yang Song, Michel Azizi, Melvin D. Lobo

**Affiliations:** 1 Barts NIHR Biomedical Research Centre, William Harvey Research Institute, Queen Mary University of London, London, UK; 2 Nephrology and Hypertension, University Hospital Erlangen, Friedrich Alexander University, Erlangen, Germany; 3 Columbia University Medical Center/New York-Presbyterian Hospital and the Cardiovascular Research Foundation, New York, NY USA; 4 Institute for Medical Engineering and Science, Massachusetts Institute of Technology, Cambridge, MA USA; 5 Klinik für Innere Medizin III, Saarland University Hospital, Homburg/Saar, Germany; 6 Department of Cardiology, Erasmus MC, University Medical Center Rotterdam, Rotterdam, NL The Netherlands; 7 Seinsheimer Cardiovascular Health Program, Medical University of South Carolina, Ralph H Johnson VA Medical Center, Charleston, SC USA; 8 Heart Center Leipzig, University of Leipzig, Leipzig, Germany; 9 Hôpital Saint-André – CHU, Bordeaux, France; 10 Deborah Heart & Lung Center, Brown Mills, NJ USA; 11 The Brigham and Women’s Hospital, Boston, MA USA; 12 University Clinic Dusseldorf, Dusseldorf, Germany; 13 Department of Cardiovascular Medicine, Princess Grace Hospital, Monaco, Monaco; 14 University Medical Center Utrecht, Utrecht, The Netherlands; 15 Minneapolis Heart Institute, Abbott Northwestern Hospital, Minneapolis, MN USA; 16 Division of Cardiovascular Medicine, State University of New York, Downstate Medical Center, New York, NY USA; 17 University Hospital of Wales, Cardiff, UK; 18 University of Exeter, Exeter, UK; 19 Department of Medicine, University of Nevada School of Medicine, Vascular Care, Renown Institute of Heart and Vascular Health, Reno, NV USA; 20 ReCor Medical, Inc, Palo Alto, CA USA; 21 Baim Institute for Clinical Research, Boston, MA USA; 22 Université de Paris, Paris, France; 23 Hypertension Department and DMU CARTE, AP-HP, Hôpital Européen Georges-Pompidou, Paris, France; 24 INSERM, CIC1418, Paris, France

**Keywords:** Hypertension, Medical research

## Abstract

The blood pressure (BP) lowering response to renal denervation (RDN) remains variable with about one-third of patients not responding to ultrasound or radiofrequency RDN. Identification of predictors of the BP response to RDN is needed to optimize patient selection for this therapy. This is a post-hoc analysis of the RADIANCE-HTN SOLO study. BP response to RDN was measured by the change in daytime ambulatory systolic blood pressure (dASBP) at 2 months post procedure. Univariate regression was used initially to assess potential predictors of outcome followed by multivariate regression analysis. In the univariate analysis, predictors of response to RDN were higher baseline daytime ambulatory diastolic blood pressure (dADBP), the use of antihypertensive medications at screening, and presence of orthostatic hypertension (OHTN) whilst the presence of untreated accessory arteries was a negative predictor of response. Multivariate analysis determined that dADBP and use of antihypertensive medications were predictors of response to RDN with a trend for OHTN to predict response. Obese females also appeared to be better responders to RDN in an interaction model. RDN is more effective in patients with elevated baseline dADBP and those with OHTN, suggesting increased peripheral vascular resistance secondary to heightened sympathetic tone. These assessments are easy to perform in clinical setting and may help in phenotyping patients who will respond better to RDN.

## Introduction

Novel antihypertensive therapies using second-generation renal denervation (RDN) catheter systems causing modification of renal sympathetic nerve signaling have shown encouraging results in randomized controlled trials [1–4]. The multicenter, randomized, double-blind, sham-controlled RADIANCE-HTN SOLO study evaluated endovascular ultrasound RDN in an off-medication population with mild–moderate combined (systolic–diastolic) hypertension [1]. To date, results from the RADIANCE-HTN SOLO study have demonstrated the efficacy and safety of the Paradise System in lowering daytime ambulatory systolic blood pressure (dASBP) in patients with primary hypertension at 2 months (off-medication) and 6 months (on-medications) post-randomization [1, 5].

Given that RDN is invasive to perform compared to drug therapy, the lack of markers of procedural success is a significant issue at the present time [6, 7]. Whilst ongoing studies are addressing potential biomarkers of successful renal nerve ablation, it would also be helpful to improve phenotyping to best determine responders/non-responders to therapy given that in RADIANCE-HTN SOLO approximately one-third of patients exhibited <5 mmHg reduction in dASBP in the treatment group at 2 months while remaining off-medications [1].

Previous studies have analyzed the clinical, anatomical, and procedural predictors of BP response to RDN [8–10]. Importantly, many of the predictors could be study specific based on factors such as the patient cohort included, study design, as well as procedural parameters including energy modality used (radiofrequency (RF) or ultrasound), number/location of ablation sites, between-interventionalist variability. Herein for the first time, we aim to analyze clinical, anatomical, and procedural predictors of BP response in a trial specific to endovascular ultrasound RDN. We specifically focused on predictors which are usually associated with sympathetically driven hypertension including higher basal heart rate [11], orthostatic hypertension [12–14], and abdominal obesity [15].

## Methods

This is a post-hoc analysis of the RADIANCE-HTN SOLO study data. To summarize, RADIANCE-HTN SOLO was a multicenter, international, double-blind, randomized, sham-controlled trial done at 21 centers in the USA and 18 centers in Europe [1]. The study was approved by local ethics committees at each center, and all participants provided written informed consent for participation in the trial.

### Patients

Male or female patients aged 18–75 years with combined systolic–diastolic hypertension (office BP ≥ 140/≥90 mmHg) on 0–2 antihypertensive medications were enrolled in the study. Patients were eligible for randomization if they had daytime ambulatory BP (ABP) ≥ 135/≥85 mmHg and <170/<105 mmHg after a 4-week discontinuation of their antihypertensive medications and had suitable renal artery anatomy on CT- or MR-angiography.

### Trial procedure

Eligible patients were randomized in a 1:1 ratio to either the sham procedure (only renal angiography) or ultrasound RDN with the Paradise catheter System (ReCor Medical, Palo Alto, CA, USA) after renal angiography under conscious sedation. Patients, outcome assessors, and study personnel were blinded to the randomization. Patients were not started on any antihypertensive medications for 2 months post procedure unless specified BP escape criteria were met.

Patients were assessed at 2 months post procedure for evaluation of the primary efficacy endpoint which was change in daytime ambulatory systolic BP (dASBP) from baseline in the intention-to-treat population. Major adverse events and safety outcomes were also collected. The study is registered with ClinicalTrials.gov, number NCT02649426.

### Office BP measurements

Office BP was recorded using the Omron^®^ MIT ELITE Plus or M10-IT device (Omron Co., Kyoto, Japan). All efforts were made to standardize office BP recordings for each patient including measurement from the same arm at same time of day using the same device by the same person. Caffeine, exercise, and smoking were avoided for at least 30 min prior to the measurement and patients sat quietly in a chair for at least 5 min before recordings.

An appropriately sized cuff was used to ensure the bladder within the BP cuff encircled at least 80% of the arm. BP was measured in both arms and the arm with the higher reading was used for office BP recording. For study defined BP measurements, three sitting BP and heart rate (HR) measurements were recorded, 1–2 min apart. The average of the 2nd and the 3rd measurements have been used for analysis. Standing BP and HR were recorded after 1 min to check the presence of OHTN, defined as office standing minus office seated systolic blood pressure (SBP) ≥ 20 mmHg and/or office standing minus office sitting diastolic blood pressure (DBP) ≥ 10 mmHg at baseline. The presence of any symptoms was not required for the diagnosis of OHTN.

### Ambulatory BP measurements

The 24-h ambulatory BP (24-H ABP) recordings were made using the Microlife^®^ WatchBP machine (Microlife, Taipei, Taiwan) at baseline and 2-month follow-up visits. The ambulatory blood pressure (ABP) was recorded on patient’s non-dominant arm for 25 h. BP was recorded every 20 min during daytime (07:00–22:00 h) and every 30 min overnight (22:00–07:00 h). 24-H ABP recordings with a minimum of 21 measurements during the daytime period were considered valid. In the case of a non-valid measurement, a new ABP recording was performed. All ABP recordings were sent to a core laboratory (dabl, Dublin, Ireland) with treatment assignments masked.

### Statistical analysis

All analyses were conducted on the per-protocol population (RDN = 64 and Sham = 58). As previously described, the per-protocol population excluded patients who did not meet baseline daytime ambulatory SBP or DBP or renal artery anatomical inclusion criteria, patients in the RDN group who did not receive bilateral RDN, patients who were treated with antihypertensive medications before the 2-month 24-H ABP, and patients who did not complete the 2-month 24-H ABP assessment [1].

Univariate regression was used to assess potential predictors of outcome defined as change in dASBP at 2 months. In addition, we evaluated whether predictors of outcome differed between responders (defined as reduction in dASBP ≥ 5 mmHg) and super responders (defined as reduction in dASBP ≥ 15 mmHg) at 2 months following treatment.

Multivariate regression analysis was conducted for the RDN arm including variables with *p* value less than 0.20 from the univariate analysis. Backward selection with a stay criterion of 0.20 was used to select predictors. In the multivariate regression analysis, we checked that the variance inflation factor was less than 5 for all input variables.

Multiple interaction analyses were conducted looking at the impact of these variables: treatment (RDN vs. Sham), sex (male vs. female), abdominal obesity (yes vs. no), and age (<55 vs. ≥ 55) on change in dASBP. The least square means were used to compare the effect size of different combinations.

Treatment effects (change in dASBP parameters, HR) were assessed comparing patients with and without orthostatic hypertension (OHTN) using analysis of covariance, adjusting for baseline values.

Abdominal obesity was defined as abdominal circumference >102 cm in males and >88 cm in females measured at the level of umbilicus. Pulse Pressure was defined as the difference between office SBP and DBP or between 24-H ambulatory SBP and DBP.

Comparisons between groups were made using two-sample *t*-tests for continuous variables and Fisher’s exact test, for categorical variables. Continuous variables are expressed as mean ± standard deviation unless otherwise specified and between-group differences are expressed as mean and corresponding two-sided 95% confidence intervals. All analyses were performed using statistical analysis system (SAS) software version 9.4 (SAS Institute, Cary, NC, US). A *p* value lower than 0.05 (two-sided) was considered significant. Reported *p* values are based on nominal values not adjusted for multiple comparisons.

## Results

### Population

The distribution of the patient population in the RADIANCE-HTN SOLO trial was evenly matched between the RDN and Sham groups and has been previously reported [1]. The baseline characteristics of the per-protocol cohort treated with endovascular ultrasound RDN included in this analysis are summarized in (Supplemental Table S1).

### Univariate analysis

All the variables assessed in the univariate model for the RDN group are listed in Table 1. The use of antihypertensive medications at screening, higher baseline dADBP, and OHTN were significant predictors, but not baseline dASBP; age, ethnicity, sex, estimated glomerular filtration rate (eGFR), hemoglobin A1C (HbA1C), body mass index (BMI), or the presence of sleep apnea. The presence of any untreated accessory artery was a negative predictor of the reduction in dASBP following RDN at 2 months. A similar univariate analysis was conducted in the Sham group and showed no variables as being significant predictors of BP response (Supplemental Table S2).

**Table 1:** Univariate analysis of predictors of response for change in daytime ambulatory systolic blood pressure from baseline to 2 months in the RDN group.

Variable	Estimate parameter	Standard error	*p* value
Age	0.1205	0.1206	0.3217
Male	0.9242	2.4923	0.7120
Black Race	1.1347	3.4717	0.7449
BMI	−0.2576	0.1994	0.2012
Abdominal obesity	−3.3343	2.4018	0.1701
Baseline eGFR	0.0651	0.0834	0.4384
Sleep apnea	−3.9201	4.1141	0.3444
Hemoglobin A1c	0.8367	1.9760	0.6734
Antihypertensive medications at screening (yes vs. no)	−6.4115	3.0965	**0.0426**
Baseline orthostatic hypertension	−6.9471	2.8698	**0.0184**
Baseline office systolic BP	0.0413	0.0936	0.6610
Baseline office diastolic BP	−0.0368	0.1510	0.8084
Baseline daytime systolic ABP	−0.0848	0.1666	0.6124
Baseline daytime diastolic ABP	−0.6148	0.2649	**0.0236**
Baseline nighttime systolic ABP	0.0117	0.0992	0.9068
Baseline nighttime diastolic ABP	−0.0340	0.1515	0.8230
24-H systolic ABP	0.0183	0.1542	0.9058
24-H diastolic ABP	−0.2926	0.2558	0.2570
Baseline office pulse pressure	0.0800	0.1122	0.4786
Baseline 24-H ambulatory pulse pressure	0.1511	0.1702	0.3781
Baseline office heart rate	−0.0570	0.0969	0.5587
Baseline 24-H ambulatory heart rate	-0.1438	0.1202	0.2361
Total number of ablations	−0.7343	1.3117	0.5777
Average main artery diameter left	0.8442	1.5629	0.5910
Average main artery diameter right	−0.5303	1.7371	0.7612
Vessel length (left renal)	0.0086	0.0986	0.9309
Vessel length (right renal)	−0.0122	0.1021	0.9055
Presence of any untreated accessory	5.4188	2.7034	**0.0494**
Presence of side branches proximal to ablations	−4.3473	3.4306	0.2098
Farthest distance bilaterally from distal ablation to parenchyma	0.0049	0.1290	0.9698
Operator case number	0.2189	0.8714	0.8025
Contrast volume used	−0.0149	0.0179	0.4087
Duration of procedure	−0.0262	0.0541	0.6296
Fluoro time	0.0799	0.2078	0.7019
Paradise catheter time	0.0967	0.0681	0.1604
Number of different balloon sizes used in a patient	−1.7074	1.6204	0.2961

*ABP* ambulatory blood pressure, *BMI* body mass index, *BP* blood pressure, *eGFR* estimated glomerular filtration rate.

Statistically significant *p*-values are in bold.

In responders to RDN (defined as reduction in dASBP ≥ 5 mmHg), the age and the presence of OHTN were positive predictors of response and the presence of any untreated accessories was a negative predictor of response. In the Sham group, there were no predictors of response identified (Table 2).

**Table 2:** Characteristics of responders (dASBP drop ≥ 5 mmHg).

	Renal denervation			Sham procedure		
Characteristics	Responder (*N* = 44)	Non-Responder (*N* = 20)	*p* value	Responder (*N* = 15)	Non-Responder (*N* = 43)	*p* value
Age (years)	52.3 ± 10.0	58.7 ± 8.7	**0.017**	51.1 ± 9.7	55.0 ± 10.2	0.202
Male	68.2% (30/44)	50.0% (10/20)	0.164	60.0% (9/15)	62.8% (27/43)	0.848
Black Race	13.6% (6/44)	15.0% (3/20)	0.884	20.0% (3/15)	16.3% (7/43)	0.743
BMI (kg/m^2^)	30.0 ± 5.8	29.5 ± 6.7	0.733	29.8 ± 6.8	29.7 ± 4.4	0.953
Abdominal obesity^a^	59.1% (26/44)	47.4% (9/19)	0.390	53.3% (8/15)	69.8% (30/43)	0.249
Baseline eGFR (ml/min/1.73 m^2^)^a^	82.2 ± 13.3	86.4 ± 16.9	0.288	86.3 ± 17.4	82.1 ± 13.7	0.343
Sleep apnea	11.4% (5/44)	5.0% (1/20)	0.418	13.3% (2/15)	11.6% (5/43)	0.861
Hemoglobin A1c (%)^a^	5.4 ± 0.7	5.5 ± 0.4	0.641	5.6 ± 0.9	5.5 ± 0.5	0.699
Antihypertensive Medications at Screening (yes vs. no)	86.4% (38/44)	75.0% (15/20)	0.264	66.7% (10/15)	79.1% (34/43)	0.334
Baseline orthostatic hypertension	27.3% (12/44)	5.0% (1/20)	**0.040**	20.0% (3/15)	16.3% (7/43)	0.743
Baseline office systolic BP (mmHg)	153.6 ± 11.9	157.0 ± 15.1	0.341	146.0 ± 14.1	154.6 ± 15.8	0.068
Baseline office diastolic BP (mmHg)	99.8 ± 7.2	100.3 ± 9.8	0.827	94.2 ± 8.5	99.6 ± 9.8	0.062
Baseline daytime systolic ABP (mmHg)	150.5 ± 7.7	150.5 ± 6.5	0.995	148.0 ± 8.6	150.1 ± 10.3	0.472
Baseline daytime diastolic ABP (mmHg)	93.9 ± 4.7	92.5 ± 3.5	0.262	93.0 ± 4.8	93.3 ± 5.6	0.855
Baseline nighttime systolic ABP (mmHg)^a^	130.5 ± 12.7	130.9 ± 11.7	0.909	130.8 ± 15.6	131.7 ± 13.2	0.828
Baseline nighttime diastolic ABP (mmHg)^a^	78.7 ± 8.4	78.4 ± 7.4	0.918	80.0 ± 7.8	80.0 ± 7.4	0.997
24-H systolic ABP (mmHg)	142.5 ± 8.4	143.6 ± 6.9	0.638	141.7 ± 10.3	143.7 ± 10.8	0.534
24-H diastolic ABP (mmHg)	87.9 ± 5.1	87.4 ± 3.9	0.691	88.2 ± 4.7	88.6 ± 5.6	0.807
Baseline office pulse pressure (mmHg)	53.8 ± 10.0	56.7 ± 12.4	0.327	51.8 ± 10.0	55.0 ± 12.4	0.374
Baseline 24-H ambulatory pulse pressure (mmHg)	54.6 ± 7.3	56.1 ± 6.7	0.432	53.5 ± 9.3	55.1 ± 9.2	0.565
Baseline office heart rate (bpm)	71.8 ± 11.9	72.9 ± 14.2	0.757	68.1 ± 8.8	71.8 ± 12.9	0.315
Baseline 24-H ambulatory heart rate (bpm)	72.6 ± 7.7	73.1 ± 14.1	0.871	71.5 ± 10.2	70.5 ± 9.8	0.725
Total number of ablations	5.5 ± 1.0	5.5 ± 0.8	0.773			
Average main artery diameter left (mm)	5.5 ± 0.7	5.4 ± 0.9	0.623			
Average main artery diameter right (mm)	5.4 ± 0.7	5.0 ± 0.7	0.053			
Vessel length (left renal) (mm)	38.0 ± 12.8	38.8 ± 11.6	0.807			
Vessel length (right renal) (mm)	43.7 ± 11.7	47.0 ± 12.4	0.317			
Presence of any untreated accessory	15.9% (7/44)	45.0% (9/20)	**0.013**			
Presence of side branches proximal to ablations	15.9% (7/44)	10.0% (2/20)	0.528			
Farthest distance bilaterally from distal ablation to parenchyma	19.2 ± 9.9	17.4 ± 8.	0.487			
Operator case number	1.9 ± 1.5	2.3 ± 1.3	0.332			
Contrast volume used (cc)^a^	145.5 ± 64.3	119.8 ± 72.6	0.168			
Duration of procedure (min)	72.0 ± 21.1	65.9 ± 25.3	0.320			
Fluoro time (min)^a^	13.0 ± 4.3	14.1 ± 8.8	0.589			
Paradise catheter time (min)	32.9 ± 18.1	33.0 ± 17.0	0.985			
Number of different balloon sizes used in patient	1.8 ± 0.8	1.5 ± 0.6	0.114			

Data displayed as % (*n*/*N*) and mean ± standard deviation.

*ABP* ambulatory blood pressure, *BMI* body mass index, *BP* blood pressure, *eGFR* estimated glomerular filtration rate.

^a^Contrast volume was missing from one patient and flouro time was missing from three patients in the renal denervation responder group. Abdominal obesity status and contrast volume each had one patient with missing data in the renal denervation non-responder group. eGFR, Nighttime SBP and Nighttime DBP each had one patient with missing data and hemoglobin A1C had two patients with missing data in the sham non-responder group.

Statistically significant *p*-values are in bold.

In super responders to RDN (defined as reduction in dASBP ≥ 15 mmHg), BMI, abdominal obesity, antihypertensive medications at screening, the presence of OHTN, baseline 24-H ambulatory heart rate (AHR), and the presence of side branches proximal to ablation, were significant predictors for BP-lowering effect of RDN. Whereas the presence of any untreated accessories was a negative predictor of response. There were no significant variables identified in the sham group (Supplemental Table S3).

### Multivariate and interaction analyses

Results of the multivariate analysis are shown in Table 3. Baseline dADBP had a small but significant impact on response to RDN with respect to the fall in dASBP. Patients taking antihypertensive medication at screening who underwent a 12-week treatment interruption had a greater response to RDN at 2 months than those who were not medicated at screening. Of note baseline dASBP/dADBP in non-medicated (*N* = 11) and medicated patients (*N* = 53) were similar at 150 ± 7.6/95 ± 4.4 mmHg and 151 ± 7.3/93 ± 4.4 mmHg, respectively. The presence of OHTN was also associated with a greater response to RDN albeit non-significant (*p* = 0.077).

**Table 3:** Multivariate analysis of predictors of response for change in daytime ambulatory systolic blood pressure from baseline to 2 months in the RDN group.

Variable	Estimate parameter	Standard error	*p* value
Antihypertensive medications at screening (yes vs. no)	−6.4952	2.9525	0.0317
Baseline orthostatic hypertension	−5.0303	2.7955	0.0770
Baseline daytime diastolic ABP	−0.5953	0.2566	0.0238

*ABP* ambulatory blood pressure.

Abdominal obesity, antihypertensive medications at screening (yes vs. no), baseline orthostatic hypertension, baseline daytime diastolic ABP, presence of any untreated accessory, and paradise catheter time were the variables included in the multivariate regression model. Then backward selection with a stay criterion of 0.20 was used to select predictors.

A variety of interaction models were analyzed (Supplemental Tables S4 and S5) with the key finding from a 3-way interaction model that females with abdominal obesity had the greatest response to RDN in the per-protocol group (Table 4). There was no difference in the BP response to RDN between obese and non-obese males and gender alone had no impact on outcome and neither did age (data not shown).

**Table 4:** Three-way interaction model of treatment arm, sex, and abdominal obesity on change in daytime ambulatory blood pressure from baseline to 2 months.

Variable	*p* value
Treatment arm	<0.001
Sex	0.967
Treatment arm × sex	0.879
Abdominal obesity	0.811
Treatment arm × abdominal obesity	0.006
Treatment arm × abdominal obesity	0.913
Treatment arm × sex × abdominal obesity	0.021

Treatment Arm	Sex	Abdominal Obesity	*n*	Change in daytime SBP at 2 M (mmHg) (least squares mean)
Renal denervation	Female	Yes	17	−12.301
Renal denervation	Female	No	6	−3.064
Renal denervation	Male	Yes	18	−7.990
Renal denervation	Male	No	22	−7.780
Sham procedure	Female	Yes	17	3.402
Sham procedure	Female	No	5	−6.315
Sham procedure	Male	Yes	21	−0.347
Sham procedure	Male	No	15	−1.846

### Patients with orthostatic hypertension

The results from the multivariate analysis suggested an influence of the presence of OHTN on ABP response to RDN in the per-protocol population. The baseline characteristics of the per-protocol RDN group were similar between patients with and without OHTN with the exception of less Caucasian ethnicity and higher baseline eGFR in patients with OHTN (Supplemental Table S6).

**Fig. 1:**
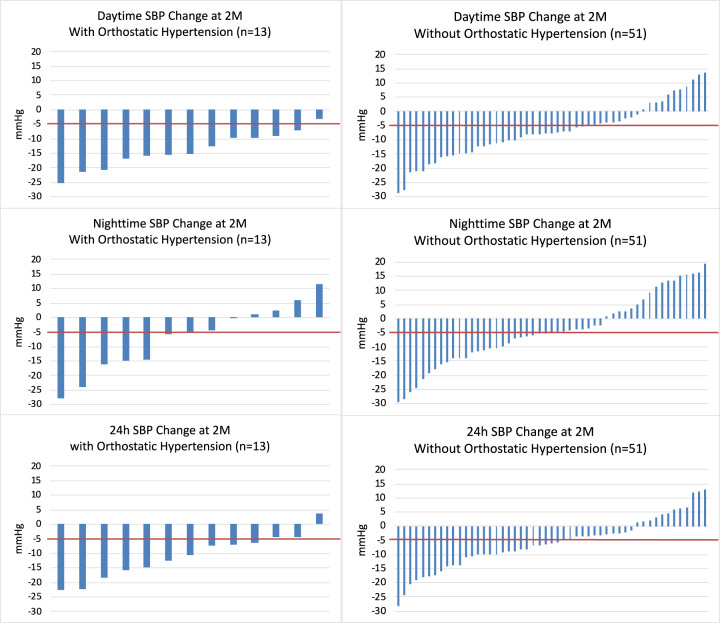
Change in SBP following RDN in individual patients with and without orthostatic hypertension. Data shown for patients with orthostatic hypertension (left panels) and without orthostatic hypertension (right panels) for daytime SBP (top panels), nighttime SBP (middle panels), and 24-H SBP (bottom panels).

The presence of OHTN at baseline (systolic or diastolic OHTN) was associated with a larger reduction in dASBP and dADBP 2 months after RDN (Table 5). Figure 1 shows that 92% (12/13) patients with OHTN demonstrated a reduction in dASBP greater than 5 mmHg, whereas 63% (32/51) patients without OHTN had such a reduction and even 22% (11/51) had a rise in dASBP. There was no influence of OHTN on the change in nighttime BP, 24-H BP, 24-H AHR or office BP or HR. Further analysis demonstrated that neither baseline office standing SBP or DBP nor the difference between baseline office standing and seated SBP or DBP were significant predictors of change in ambulatory or office BP and HR (data not shown). In the RDN patients with OHTN at baseline, 69% (9/13) no longer met the definition of OHTN at 2 months after RDN.

**Table 5:** Ambulatory BP and HR changes from baseline to 2 months for RDN patients with and without orthostatic hypertension.

Parameters	With orthostatic hypertension (*N* = 13)	Without orthostatic hypertension (*N* = 51)	*p* value
Change from baseline to 2 months			
Daytime SBP (mmHg)	−14.00 ± 6.26	−7.05 ± 9.82	0.022
Daytime DBP (mmHg)	−8.92 ± 5.10	−4.47 ± 5.88	0.025
Nighttime SBP (mmHg)	−7.07 ± 11.75	−4.24 ± 12.32	0.641
Nighttime DBP (mmHg)	−5.28 ± 8.18	−3.13 ± 9.00	0.488
24-H SBP (mmHg)	−10.98 ± 7.68	−6.01 ± 9.04	0.094
24-H DBP (mmHg)	−7.28 ± 5.55	−3.93 ± 6.01	0.111
24-H HR (bpm)	0.55 ± 4.52	1.24 ± 4.68	0.700
Office SBP (mmHg)	−12.62 ± 13.77	−9.27 ± 12.57	0.520
Office DBP (mmHg)	−3.08 ± 10.56	−5.63 ± 7.92	0.425
Office HR (bpm)	−0.31 ± 8.83	−0.82 ± 10.19	0.761

Data displayed as mean ± standard deviation.

*DBP* diastolic blood pressure, *HR* heart rate, *SBP* systolic blood pressure.

## Discussion

Previous studies have identified baseline SBP, younger age, higher eGFR, higher HR, use of central sympatholytic agents or aldosterone antagonists, indices of baseline sympathetic overdrive, and various biological markers as markers of response to catheter-based RF RDN [10, 16–22].

In addition to being limited to populations treated with RF energy to achieve renal sympathectomy, these studies mostly included patients taking antihypertensive medications. Given that dynamic patterns of adherence and non-adherence to antihypertensive drugs in trials are now established, it might be expected that this would have confounding effects on clinical outcomes [23]. The RADIANCE-HTN SOLO study thus provides us a unique opportunity to evaluate predictors of BP-lowering response to endovascular ultrasound-based RDN in a non-medicated population, eliminating thus the confounding effect of medication use.

In this post-hoc analysis of the RADIANCE-HTN SOLO trial, we found that the predictors of a larger response to RDN were mainly linked to heightened sympathetic nervous system (SNS) activity in addition to anatomical predictors. Indeed, the positive predictors of BP response to endovascular ultrasound RDN were use of antihypertensive medications at screening, higher baseline dADBP, abdominal obesity, female sex, and OHTN. Patients receiving antihypertensive medications at screening may have demonstrated an enhanced response to RDN due to medication-induced priming of the SNS at the time of screening even though baseline BPs were similar between the groups [24]. Patients with higher baseline dADBP having greater response suggests that in middle-aged patients with milder forms of combined hypertension, there may be heightened SNS activity and that increased DBP, reflecting increased systemic vascular resistance which is under neurohumoral control, might act as a surrogate marker for this [25]. Indeed, it has been recognized that whole-body sympathetic neural activity rises in both males and females after the age of 30 years [26]. Notably, and in contrast to earlier studies in patients with resistant hypertension, baseline office SBP and dASBP did not influence the response to RDN and this was also observed in the SPYRAL HTN-OFF MED study, again possibly related to the selection of patients with milder forms of hypertension in both the SPYRAL HTN-OFF MED and RADIANCE-HTN SOLO studies [1, 4]. Also, increased DBP is associated with increased systemic vascular resistance, which is more easily reversible by any antihypertensive treatment, whereas increased SBP is associated with increased aortic stiffness and structural arterial changes which are much less reversible [27].

Finally, muscle sympathetic nerve activity (MSNA) is an established surrogate marker for SNS activity. In males and females more than 40 years of age, MSNA correlates significantly with mean arterial pressure, which has a major contribution from diastolic pressure compared to systolic pressure [28]. It is also known that early HTN is characterized by increased cardiac output and established HTN is characterized by high vascular resistance [29]. Hence diastolic BP and vascular resistance are better surrogates of SNS overactivity and may therefore be better predictors of response to RDN compared to systolic BP. These findings are consistent with SPYRAL HTN-OFF MED study findings.

In the three-way interaction model, females with abdominal obesity showed striking reduction in BP following RDN. Abdominal obesity is also a known feature of the metabolic syndrome characterized by higher baseline SNS activity [30]. Moreover, the mean age of the females in our study was 54 years and increasing age in women has been demonstrated to result in striking upregulation in MSNA independent of the menopause as well as increase in BP [28]. Thus, obese females may represent a group for whom strategies targeting sympathetic regulation of the circulation may be particularly effective [31].

The presence of systolic and/or diastolic OHTN was a positive predictor of dASBP and dADBP lowering following RDN in the univariate analysis although this did not reach significance in the multivariate analysis. Interestingly, the OHTN phenotype was also associated with younger age, female sex, higher BMI, and eGFR, but the sample size may have been too small to achieve statistical significance in most factors (Supplemental Table S6).

A preliminary report from the SPYRAL HTN-OFF MED study noted OHTN as a predictor of response to RF-based RDN and notably, in both this study and herein, patients with OHTN exhibited larger BP reduction following RDN with markedly reduced variability of dASBP response compared to the overall per-protocol treatment groups [32]. Of interest, in RADIANCE-HTN SOLO only daytime BP parameters were significantly reduced in patients with OHTN with no effects on nocturnal and 24-h BP levels or HR perhaps due to reduction in SNS activation with recumbency and sleep and resultant lessened impact of sympathomodulatory treatment.

The pathophysiology of OHTN is poorly understood but it is considered a manifestation of SNS dysfunction [33]. Systolic OHTN is associated with older age and a stiff circulation, whereas diastolic OHTN (seen in the majority of our patients) is associated with higher resting cardiac output, HR and urinary norepinephrine output suggesting increased baseline SNS activity [34]. It is associated with increased activity of the SNS to the resistance vessels causing vasoconstriction and in particular increase in diastolic pressure. Notably, it was previously shown that BP lowering associated with RDN was associated with a significant reduction in total peripheral resistance [35]. Together these factors strongly suggest that in hypertensive patients with OHTN, a treatment such as RDN that reduces SNS activity might be effective and that OHTN per se may be valuable as a screening tool for neurogenic hypertension and improved response to RDN [36].

## Conclusions

In this study, the positive predictors of response to RDN were higher baseline dADBP and the use of antihypertensive medications at screening. The presence of untreated accessory arteries was a negative predictor of response to RDN in the univariate analysis without reaching significance in the multivariate analysis. The presence of OHTN did not reach significance in the multivariate analysis but is an interesting finding and needs to be explored in future studies with bigger numbers. Obese females also appeared to be better responders to RDN in the interaction model. All of the findings noted above merit exploration in larger randomized controlled trials as identifying patients most suitable for RDN remains a clinical priority given that efficacy and safety of this therapy has been established [1–4].

### Limitations of this study

This study was a post-hoc analysis of a small sample size albeit from a rigorously conducted randomized clinical trial with objective endpoints and negligible data loss. In as such, our results can only be considered as hypothesis generating. We focused on the per-protocol population to ensure minimization of confounding through contaminating effects of medication. However, we did not strictly test blood or urine for drug metabolites so unauthorized consumption of antihypertensive medications cannot be completely ruled out. On the other hand, since these findings occurred in the setting of a well-controlled trial in patients off antihypertensive medications, they may be less applicable in real-world clinical practice. Finally, OHTN is ideally recorded as the difference between lying and standing BPs rather than seated and standing. If anything, we are likely to have diagnosed less patients with OHTN as a result although every single patient that demonstrated OHTN in our study showed BP reduction with RDN.

## Summary

### What is known about this topic

- RADIANCE-HTN SOLO study results have shown significant BP reduction with endovascular ultrasound RDN albeit with variable response.
- Previous RDN studies using RF energy have looked at predictors of response in patients on BP-lowering medication.
- Predictors of response can vary with energy modality used (RF vs. ultrasound), study design, and patient population studied.

### What this study adds

- This is the first evaluation of clinical, anatomical, and procedural predictors of response specific to endovascular ultrasound RDN.
- The study design offers a unique opportunity to evaluate predictors in absence of BP-lowering drugs in the study cohort.
- The study has identified novel predictors (abdominal obesity, orthostatic hypertension) that show bigger BP response with RDN and should be evaluated in future studies.

## Supplementary information

Supplemental Materials
