# Supplementary material for: Predictors of blood pressure response to ultrasound renal denervation in the RADIANCE-HTN SOLO study
Source: J Hum Hypertens. 2021 May 24;36(7):629–39. doi: 10.1038/s41371-021-00547-y (PMC9287166; doi:10.1038/s41371-021-00547-y)
Supplement: Supplementary file 1 — Supplemental Materials [file 41371_2021_547_MOESM1_ESM.pdf]

Predictors of BP Response Supplemental Material

Tables included in supplemental materials have been provided by the authors to give readers additional information about their work.

Table S1. Baseline characteristics

Characteristic	Renal Denervation (N=64)	Sham Procedure (N=58)
Age (years)	54.3±10.0	54.0±10.2
Female sex	37.5% (24/64)	37.9% (22/58)
Race		
White	82.8% (53/64)	72.4% (42/58)
Black	14.1% (9/64)	17.2% (10/58)
Other	3.1% (2/64)	10.3% (6/58)
Body mass index – kg/m^2	29.9±6.0	29.7±5.0
Abdominal obesity*	55.6% (35/63)	65.5% (38/58)
eGFR – $ml/min/1.73m^{2*}$	83.5±14.5	83.2±14.8
eGFR <60 $ml/min/1.73m^2$	1.5% (1/64)	1.8% (1/57)
Type 2 diabetes	3.1% (2/64)	8.6% (5/58)
Sleep apnea	9.4% (6/64)	12.1% (7/58)
**Seated office blood pressure and heart rate prior to antihypertensive medication washout**		
SBP (mmHg)	142.6±14.8	145.2±15.3
DBP (mmHg)	91.9±10.0	94.2±8.2
Heart rate (bpm)*	73.2±12.0	71.2±11.8
**Number of Antihypertensive medications at screening**		
0	17.2% (11/64)	24.1% (14/58)
1 medication	39.1% (25/64)	39.7% (23/58)

2 medications in combination	42.2% (27/64)	36.2% (21/58)
3 medications in combination	1.6% (1/64)	0.0% (0/58)
**Baseline ambulatory blood pressure following antihypertensive medication washout**		
Daytime SBP (mmHg)	150.5±7.3	149.5±9.9
Daytime DBP (mmHg)	93.5±4.4	93.2±5.4
Nighttime SBP (mmHg)*	130.6±12.3	131.5±13.7
Nighttime DBP (mmHg)*	78.6±8.0	80.0±7.4
24h SBP (mmHg)	142.9±7.9	143.2±10.6
24h DBP (mmHg)	87.8±4.7	88.5±5.4

Data displayed as % (n/N) and mean ± standard deviation.

DBP: diastolic blood pressure; eGFR: estimated glomerular filtration rate; SBP: systolic blood pressure;.

* Abdominal obesity status and office heart rate prior to medication washout each had one patient with missing data in the renal denervation group. eGFR, Nighttime SBP and Nighttime DBP each had one patient with missing data in the sham arm.

Table S2. Univariate analysis of predictors of response for change in daytime ambulatory systolic blood pressure from baseline to 2 months in the Sham group

Variable	Estimate Parameter	Standard Error	P value
Age	0.0845	0.1109	0.4493
Male	-2.8058	2.2815	0.2239
Black Race	1.8429	2.9597	0.5360
BMI	0.2305	0.2224	0.3044
Abdominal Obesity	4.5024	2.2823	0.0535
Baseline eGFR	-0.0171	0.0775	0.8266
Sleep Apnea	-2.6261	3.4258	0.4466
Hemoglobin A1c	-0.8569	1.7703	0.6303
Antihypertensive Medications at Screening (yes vs. no)	0.1344	2.6216	0.9593
Baseline Orthostatic hypertension	0.8521	2.9677	0.7751
Baseline Office SBP	0.1096	0.0706	0.1260
Baseline Office DBP	0.1872	0.1140	0.1061
Baseline Daytime Systolic ABP	-0.1330	0.1133	0.2456
Baseline Daytime Diastolic ABP	-0.2139	0.2094	0.3115
Baseline Nighttime Systolic ABP	-0.0690	0.0834	0.4114
Baseline Nighttime Diastolic ABP	-0.0609	0.1550	0.6959
24-hour Ambulatory Systolic Blood Pressure	-0.1170	0.1052	0.2709
24-hour Ambulatory Diastolic Blood Pressure	-0.1777	0.2099	0.4009
Baseline Office Pulse Pressure	0.0675	0.0954	0.4822
Baseline 24h ABPM Pulse Pressure	-0.0960	0.1222	0.4352

Variable	Estimate Parameter	Standard Error	P value
Baseline Office Heart Rate	0.0483	0.0938	0.6088
Baseline 24h Ambulatory Heart Rate	-0.0409	0.1151	0.7237

ABP: ambulatory blood pressure; BMI: body mass index; eGFR: estimated glomerular filtration rate

Table S3. Characteristics of super-responders (dASBP drop ≥15mmHg)

	**Renal Denervation**			**Sham Procedure**		
**Characteristics**	**Responder (N=17)**	**Non-Responder (N=47)**	**p-value**	**Responder (N=3)**	**Non-Responder (N=55)**	**p-value**
Age (years)	55.1±7.9	54.0±10.7	0.702	56.0±8.7	53.9±10.3	0.725
Male	47.1% (8/17)	68.1% (32/47)	0.125	66.7% (2/3)	61.8% (34/55)	0.866
Black Race	11.8% (2/17)	14.9% (7/47)	0.750	0.0% (0/3)	18.2% (10/55)	0.417
BMI (kg/m^2)	32.5±5.7	28.9±5.9	**0.036**	27.6±4.4	29.8±5.1	0.465
Abdominal Obesity	82.4% (14/17)	45.7% (21/46)	**0.009**	66.7% (2/3)	65.5% (36/55)	0.966
Baseline eGFR (ml/min/1.73m^2)	84.2±17.6	83.3±13.5	0.830	81.7±22.0	83.3±14.6	0.860
Sleep Apnea	17.6% (3/17)	6.4% (3/47)	0.172	33.3% (1/3)	10.9% (6/55)	0.246
HbA1c (%)	5.5±0.6 (17)	5.5±0.6 (47)	0.792	5.8±0.9	5.5±0.6	0.580
Antihypertensive Medications at Screening (yes vs. no)	100.0% (17/17)	76.6% (36/47)	**0.028**	66.7% (2/3)	76.4% (42/55)	0.702
Baseline Orthostatic hypertension	41.2% (7/17)	12.8% (6/47)	**0.013**	0.0% (0/3)	18.2% (10/55)	0.417
Baseline Office SBP (mmHg)	154.4±10.5	154.8±13.9	0.902	137.3±16.6	153.2±15.4	0.089

	Renal Denervation			Sham Procedure		
**Characteristics**	**Responder (N=17)**	**Non-Responder (N=47)**	**p-value**	**Responder (N=3)**	**Non-Responder (N=55)**	**p-value**
Baseline Office DBP (mmHg)	98.9±7.0	100.3±8.4	0.544	93.3±9.5	98.5±9.7	0.376
Baseline Daytime Systolic ABP (mmHg)	150.8±7.5	150.4±7.3	0.831	152.1±13.9	149.4±9.8	0.645
Baseline Daytime Diastolic ABP (mmHg)	94.9±4.9	93.0±4.1	0.129	94.5±3.8	93.2±5.4	0.689
Baseline Nighttime Systolic ABP (mmHg)	132.8±13.3	129.8±11.9	0.388	141.7±23.1	130.9±13.2	0.189
Baseline Nighttime Diastolic ABP (mmHg)	81.2±9.6	77.6±7.3	0.120	84.3±6.6	79.7±7.4	0.302
24-hour Ambulatory Systolic Blood Pressure (mmHg)	143.5±8.0	142.6±7.9	0.684	148.3±17.7	142.9±10.3	0.399
24-hour Ambulatory Diastolic Blood Pressure (mmHg)	89.4±5.7	87.2±4.2	0.100	90.6±5.1	88.4±5.4	0.480

	Renal Denervation			Sham Procedure		
**Characteristics**	**Responder (N=17)**	**Non-Responder (N=47)**	**p-value**	**Responder (N=3)**	**Non-Responder (N=55)**	**p-value**
Baseline Office Pulse Pressure (mmHg)	55.4±10.4	54.5±11.0	0.760	44.0±7.5	54.7±11.8	0.127
Baseline 24h ABPM Pulse Pressure (mmHg)	54.2±6.4	55.4±7.4	0.531	57.6±13.2	54.5±9.1	0.575
Baseline Office Heart Rate (bpm)	75.2±10.9	71.1±13.0	0.252	68.3±4.2	71.0±12.3	0.714
Baseline 24h Ambulatory Heart Rate (bpm)	76.6±5.6	71.4±10.9	**0.017**	75.1±10.2	70.5±9.8	0.434
Total Number of Ablations	5.6±0.9	5.4±0.9	0.449			
Average Main Artery Diameter L (mm)	5.2±0.7	5.6±0.8	0.067			
Average Main Artery Diameter R (mm)	5.0±0.6	5.3±0.7	0.068			
Vessel Length (L Renal) (mm)	37.4±10.2	38.5±13.1	0.751			
Vessel Length (R Renal) (mm)	46.9±10.0	43.9±12.5	0.387			
Presence of Any Untreated Accessory	5.9% (1/17)	31.9% (15/47)	**0.034**			

	Renal Denervation			Sham Procedure		
**Characteristics**	**Responder (N=17)**	**Non-Responder (N=47)**	**p-value**	**Responder (N=3)**	**Non-Responder (N=55)**	**p-value**
Presence of Side branches Proximal to Ablations	29.4% (5/17)	8.5% (4/47)	**0.034**			
Farthest Distance Bilaterally from Distal Ablation to Parenchyma	18.9±9.6	18.6±9.5	0.907			
Operator Case #	2.1±1.7	2.0±1.3	0.968			
Contrast volume used (cc)*	123.4±52.0	143.0±72.2	0.309			
Duration of procedure (min)	70.6±18.3	69.9±24.0	0.904			
Fluoro time (min)*	13.1±2.4	13.5±7.0	0.742			
Paradise Catheter time (min)	27.5±13.6	34.9±18.6	0.136			
Number of Different Balloon Sizes Used in Patient	1.8±0.8	1.7±0.7	0.769			

Data displayed as % (n/N) and mean ± standard deviation.
ABP: ambulatory blood pressure ; BMI: body mass index; eGFR: estimated glomerular filtration rate
* Flouro time was missing from one patient in the renal denervation responder group. Abdominal obesity status had one patient with missing data and contrast volume and flouro time each had two patients with missing data in the renal denervation non-responder group. Baseline eGFR, Nighttime SBP and Nighttime DBP each had one patient with missing data and HbA1c had two subjects with missing data in the sham non-responder group.

Table S4. Three-way interaction model of treatment arm, sex, and age on change in daytime ABPM from baseline to 2 months

**Variable**	**p-value**
Treatment Arm	<0.001
Sex	0.878
Treatment Arm * Sex	0.246
Age	0.081
Treatment Arm*Age	0.335
Sex*Age	0.465
Treatment Arm*Sex*Age	0.325

**Treatment Arm**	**Sex**	**Age** (years)	**n**	**Change in Daytime SBP at 2M** (Least Squares Mean)
Renal Denervation	Female	≥55	13	-5.827
Renal Denervation	Female	<55	11	-13.418
Renal Denervation	Male	≥55	21	-7.048
Renal Denervation	Male	<55	19	-8.774
Sham Procedure	Female	≥55	10	1.656
Sham Procedure	Female	<55	12	0.717
Sham Procedure	Male	≥55	20	-0.170
Sham Procedure	Male	<55	16	-1.968

Table S5. Four-way interaction model of treatment arm, sex, age, and abdominal obesity on change in daytime ABPM from baseline to 2 months

**Variable**	**p-value**
Treatment Arm	0.002
Sex	0.551
Treatment Arm*Sex	0.904
Age	0.281
Treatment Arm*Age	0.087
Sex*Age	0.733
Treatment Arm*Sex*Age	0.120
Abdominal Obesity	0.483
Treatment Arm*Abdominal Obesity	0.015
Sex*Abdominal Obesity	0.696
Treatment Arm*Sex*Abdominal Obesity	0.023
Age*Abdominal Obesity	0.504
Treatment Arm*Age*Abdominal Obesity	0.249
Sex*Age*Abdominal Obesity	0.795
Treatment Arm*Sex*Age*Abdominal Obesity	0.927

**Treatment Arm**	**Sex**	**Age** (years)	**Abdominal Obesity**	**n**	**Change in Daytime SBP at 2M** (Least Squares Mean)
Renal Denervation	Female	≥55	Yes	9	-8.674
Renal Denervation	Female	≥55	No	4	0.485
Renal Denervation	Female	<55	Yes	8	-16.511
Renal Denervation	Female	<55	No	2	-10.749
Renal Denervation	Male	≥55	Yes	7	-6.938
Renal Denervation	Male	≥55	No	14	-6.945
Renal Denervation	Male	<55	Yes	11	-8.516
Renal Denervation	Male	<55	No	8	-9.061
Sham Procedure	Female	≥55	Yes	9	3.090
Sham Procedure	Female	≥55	No	1	-12.131
Sham Procedure	Female	<55	Yes	8	3.471
Sham Procedure	Female	<55	No	4	-5.053
Sham Procedure	Male	≥55	Yes	12	1.937
Sham Procedure	Male	≥55	No	8	-3.075
Sham Procedure	Male	<55	Yes	9	-3.295
Sham Procedure	Male	<55	No	7	-0.250

Table S6. Baseline characteristics of subjects with and without orthostatic hypertension

Characteristics	With orthostatic hypertension (N=13)	Without orthostatic hypertension (N=51)	p-value
Age (years)	52.5±13.1	54.8±9.2	0.479
Female	46.2% (6/13)	35.3% (18/51)	0.470
Race			
White	69.2% (9/13)	86.3% (44/51)	0.016
Black	15.4% (2/13)	13.7% (7/51)	
Other	15.4% (2/13)	0.0% (0/51)	
BMI (kg/m^2)	32.5±7.0	29.2±5.7	0.084
Abdominal Obesity*	76.9% (10/13)	50.0% (25/50)	0.082
eGFR ($ml/min/1.73m^2$)	90.6±18.6	81.7±12.9	0.046
eGFR<60	100.0% (13/13)	98.0% (50/51)	0.611
Type 2 Diabetes	7.7% (1/13)	2.0% (1/51)	0.289
Sleep apnea	0.0% (0/13)	11.8% (6/51)	0.194
**Seated office blood pressure and heart rate prior to antihypertensive medication washout**			
SBP (mmHg)	145.2±15.9	142.0±14.6	0.480
DBP (mmHg)	91.0±10.4	92.1±10.0	0.722
Heart rate (bpm)*	72.3±10.0	73.4±12.5	0.786
**Number of Antihypertensive medications at screening**			
0	7.7% (1/13)	19.6% (10/51)	0.416
1 medication	30.8% (4/13)	41.2% (21/51)	

Characteristics	With orthostatic hypertension (N=13)	Without orthostatic hypertension (N=51)	p-value
2 medications in combination	61.5% (8/13)	37.3% (19/51)	
3 medications in combination	0.0% (0/13)	2.0% (1/51)	
**Baseline ambulatory blood pressure following antihypertensive medication washout**			
Daytime SBP (mmHg)	152.8±6.6	149.9±7.4	0.199
Daytime DBP (mmHg)	95.0±4.2	93.1±4.4	0.163
Nighttime SBP (mmHg)	132.8±13.1	130.1±12.1	0.481
Nighttime DBP (mmHg)	79.3±7.7	78.4±8.2	0.727
24h SBP (mmHg)	144.8±8.0	142.4±7.9	0.321
24h DBP (mmHg)	88.9±4.8	87.5±4.7	0.355

Data displayed as % (n/N) and mean ± standard deviation.
BMI: body mass index; DBP: diastolic blood pressure; eGFR: estimated glomerular filtration rate; SBP: systolic blood pressure
*One subject in the sham arm is missing abdominal obesity status and one subject in the RDN arm is missing screening office heart rate.
